# Outpatient Antibiotic Use and Treatment Failure Among Children With Pneumonia

**DOI:** 10.1001/jamanetworkopen.2024.41821

**Published:** 2024-10-29

**Authors:** Daniel J. Shapiro, Matt Hall, Mark I. Neuman, Adam L. Hersh, Jillian M. Cotter, Jonathan D. Cogen, Thomas V. Brogan, Lilliam Ambroggio, Anne J. Blaschke, Susan C. Lipsett, Jeffrey S. Gerber, Todd A. Florin

**Affiliations:** 1 Division of Pediatric Emergency Medicine, University of California, San Francisco; 2 Children’s Hospital Association, Lenexa, Kansas; 3 Division of Emergency Medicine, Boston Children’s Hospital, Boston, Massachusetts; 4 Division of Pediatric Infectious Diseases, University of Utah, Salt Lake City; 5 Section of Hospital Medicine, University of Colorado and Children’s Hospital Colorado, Aurora; 6 Division of Pulmonary Medicine and Sleep Medicine, Seattle Children’s Hospital, Seattle, Washington; 7 Division of Critical Care Medicine, Seattle Children’s Hospital, Seattle, Washington; 8 Section of Emergency Medicine, Children’s Hospital Colorado, Aurora; 9 Division of Infectious Diseases, Children’s Hospital of Philadelphia, Philadelphia, Pennsylvania; 10 Division of Emergency Medicine, Ann & Robert H. Lurie Children’s Hospital, Chicago, Illinois; 11 Department of Pediatrics, Northwestern University Feinberg School of Medicine, Chicago, Illinois

## Abstract

**Importance:**

Although a viral etiology can be detected in most cases of pediatric pneumonia, antibiotic treatment is common. The effectiveness of antibiotics in the outpatient setting for children diagnosed with pneumonia is not known.

**Objective:**

To compare outcomes among children diagnosed with pneumonia who were and were not treated with oral antibiotics.

**Design, Setting, and Participants:**

This retrospective cohort study identified Medicaid-insured children and adolescents 17 years or younger diagnosed with pneumonia and discharged from ambulatory settings in a multistate claims database from January 1, 2017, to December 31, 2019. Propensity score matching was used to mitigate confounding. Data were analyzed from August 31, 2023, to August 16, 2024.

**Exposures:**

Antibiotic receipt, defined as an oral antibiotic dispensed from a pharmacy on the day of the index visit or on the subsequent day. Children who did not receive antibiotics included those who were not prescribed antibiotics and those who were prescribed antibiotics but did not fill the prescription.

**Main Outcomes and Measures:**

Treatment failure and severe outcomes within 2 to 14 days after the index visit. Treatment failure included hospitalization or ambulatory revisits for pneumonia, new antibiotic dispensation with a same-day ambulatory visit, or complicated pneumonia. Severe outcomes included hospitalization for pneumonia or complicated pneumonia.

**Results:**

Among the 103 854 children with pneumonia included in the analysis, the median age was 5 (IQR, 2-9) years, and 54 665 (52.6%) were male. Overall, 20 435 children (19.7%) did not receive an antibiotic within 1 day. The propensity score–matched analysis included 40 454 children (20 227 per group). Treatment failure occurred in 2167 children (10.7%) who did not receive antibiotics and 1766 (8.7%) who received antibiotics (risk difference, 1.98 [95% CI, 1.41-2.56] percentage points). Severe outcomes occurred in 234 of 20 435 children (1.1%) who did not receive antibiotics and in 133 of 83 419 (0.7%) who did (risk difference, 0.46 [95% CI, 0.28-0.64] percentage points).

**Conclusions and Relevance:**

In this cohort study of children diagnosed with pneumonia in ambulatory settings, almost 20% did not receive antibiotics within a day of diagnosis. Although not receiving antibiotics was associated with a small increase in the risk of treatment failure, severe outcomes were uncommon regardless of whether antibiotics were received. These results suggest that some children diagnosed with pneumonia can likely be managed without antibiotics and highlight the need for prospective studies to identify these children.

## Key Points

**Question:**

In children with pneumonia managed as outpatients, is receipt of antibiotics associated with decreased risk of treatment failure?

**Findings:**

In this cohort study of 103 854 children with pneumonia treated in an outpatient setting, 20% did not receive antibiotics. Treatment failure was uncommon (8.7% received antibiotics and 10.7% did not receive antibiotics), and severe outcomes were rare, regardless of whether antibiotics were received (0.7% receiving antibiotics and 1.1% not receiving antibiotics).

**Meaning:**

These findings suggest that future efforts to identify children with pneumonia who can safely be managed without antibiotics are warranted.

## Teaser

This cohort study examines the frequency with which children diagnosed with pneumonia in outpatient settings do not receive antibiotics and compares the risk of treatment failure and severe outcomes between children who receive antibiotics and those who do not.

## Introduction

Pneumonia is diagnosed during 1.5 million ambulatory care visits each year among children in the US.^1^ Because viral pathogens can be detected in most cases,^2,3^ especially in younger children, guidelines from the Infectious Diseases Society of America recommend that preschool-aged children with pneumonia who are well enough to be treated in an outpatient setting do not routinely require antibiotic treatment.^4^ However, in practice, antibiotics are prescribed for most children,^1,5,6^ likely because of concerns about bacterial coinfection and lack of published evidence to guide a decision not to prescribe antibiotics.^2^ Treating children with antibiotics when they are unlikely to provide benefit contributes to antibiotic resistance and exposes children to potential risks, including rash, diarrhea, mislabeling of allergy, and, rarely, severe complications.^7,8,9^

In children outside of the US with nonsevere, so-called fast-breathing pneumonia as defined by the World Health Organization, randomized clinical trials have shown only modest effects of amoxicillin compared with placebo.^10,11^ One study of 337 children with pneumonia and other lower respiratory tract infections discharged from a single emergency department (ED) in the US^12^ found no association of antibiotics with treatment failure. However, no large-scale studies in the US have compared clinical outcomes between children diagnosed with pneumonia in an outpatient setting who were treated with and without antibiotics.

The objectives of this study were to identify the frequency with which children diagnosed with pneumonia in outpatient settings do not receive antibiotics and to compare the risk of treatment failure and severe outcomes between children who receive antibiotics and those who do not. A clearer understanding of the natural history of untreated pneumonia and the comparative effectiveness of antibiotic treatment for pneumonia may inform future studies that identify which children with nonsevere pneumonia can be managed safely without antibiotics.

## Methods

### Design and Data Source

We performed a retrospective cohort study using data from the Merative MarketScan Medicaid database from January 1, 2017, through December 31, 2019.^13^ The database includes insurance claims from hospitals, ambulatory care settings, and pharmacies for more than 6 million enrollees in 7 to 10 anonymized states. We chose a period predating 2020—the most recent year in which the data were available to us—because of known changes to the circulating respiratory pathogens and patterns of use of health care services during the early stages of the COVID-19 pandemic.^14^ As this study included deidentified data, it was considered non–human participants research by the Ann & Robert H. Lurie Children’s Hospital, Chicago, Illinois, Institutional Review Board and was therefore considered exempt from informed consent. We followed the Strengthening the Reporting of Observational Studies in Epidemiology (STROBE) guideline.

### Setting and Participants

We included encounters by children and adolescents (hereinafter referred to as children) aged 1 month to 17 years who were discharged from ambulatory care settings with a diagnosis of pneumonia. For children who had multiple visits for pneumonia during the study period, we considered only the first eligible visit. We defined ambulatory care settings as offices, urgent care settings, or emergency departments according to *Current Procedural Terminology* and Centers for Medicare & Medicaid Services place of service codes^15^ (eTable 1 in Supplement 1). We defined pneumonia according to *International Statistical Classification of Diseases, Tenth Revision, Clinical Modification* codes J13, J14, J15, and J18 assigned at the visit.

To ensure identification of comorbidities and clinical outcomes, we excluded children who were not continuously enrolled in Medicaid for 6 months before and 14 days after the index visit. We also excluded children who were hospitalized during the 30 days prior to the index visit to avoid the inclusion of children with hospital-acquired pneumonia. To ensure accurate assignment of the primary exposure (antibiotic treatment), we excluded children who received antibiotics during the 14 days prior to the index visit or who were treated with intravenous antibiotics during the index visit. Similarly, we excluded children who received an antibiotic for fewer than 3 days or more than 14 days, as such prescriptions were unlikely to be for community-acquired pneumonia. Finally, we excluded children for whom a concomitant diagnosis of a bacterial infection potentially warranting antibiotics (eg, urinary tract infection) was made at the index visit, as the concomitant infection may have been the target of the antibiotic therapy and may have contributed to the outcomes experienced by these children independent of antibiotic treatment. These concomitant diagnoses were identified by independent manual review of diagnosis codes by 2 authors (D.J.S. and T.A.F.). Administrative codes are listed in eTable 1 in Supplement 1.

### Exposure

The primary exposure was receipt of oral antibiotics on the day of the index visit or on the subsequent day. Antibiotic receipt was defined as the presence of a claim for an antibiotic dispensation (ie, a fill) from a pharmacy. Because claims data do not contain information about whether a clinician provided an antibiotic prescription, children who did not receive antibiotics by our definition included those who were not prescribed an antibiotic and those who were prescribed an antibiotic but did not fill the prescription. A 2-day exposure window was chosen to reflect immediate (rather than delayed) treatment. This approach identified 98% of antibiotics received within 7 days after the index visit. Antibiotics included any of the first- or second-line recommended treatments for outpatient pneumonia according to Infectious Diseases Society of America guidelines^4^ (eTable 1 in Supplement 1).

### Outcomes

The primary outcome was treatment failure during the 2 to 14 days after the index visit, defined as a composite outcome consisting of any of the following: (1) hospitalization with a diagnosis of pneumonia; (2) ED or urgent care visit with a diagnosis of pneumonia; (3) antibiotic dispensation with a same-day visit to an ED, urgent care center, or outpatient clinic; or (4) a diagnosis of complicated pneumonia.^16^ We considered antibiotic dispensations with same-day visits to capture new antibiotics dispensed after instruction by a clinician during a follow-up visit, rather than prescriptions prescribed at the index encounter that were filled after a time delay. Complicated pneumonia was identified using *Current Procedural Terminology* and *International Statistical Classification of Diseases, Tenth Revision, Clinical Modification* codes and included a pleural drainage procedure or a diagnosis of a parapneumonic effusion, empyema, necrotizing pneumonia, or lung abscess^17^ (eTable 1 in Supplement 1).

Secondary outcomes included any severe outcome, defined as hospitalization with a diagnosis of pneumonia or complicated pneumonia. Additional secondary outcomes included all-cause hospitalizations, all-cause revisits, and antibiotic fills (regardless of whether a same-day visit occurred). All secondary outcomes were measured during the 2 to 14 days after the index visit.

### Statistical Analysis

Data were analyzed from August 31, 2023, to August 16, 2024. For comparisons of proportions across exposure groups before propensity matching, we used χ^2^ test, and the McNemar test was used after matching. To adjust for potential confounding across exposure groups, we performed 1:1 propensity matching with a nearest neighbor approach using a greedy algorithm with a caliper set at 0.1 of the SD of the logit of the propensity score and matching that minimized the difference between the logits of the propensity scores. Variables used to create propensity scores were chosen based on an a priori assessment of their potential to affect random assignment of the exposure and included age group (1-4, 5-12, and 13-17 years), race and ethnicity (Hispanic, non-Hispanic Black, non-Hispanic White, other [including non-Hispanic ethnicities with races other than Black or White], or missing), a complex chronic condition^18^ diagnosed within the prior 6 months (yes or no), a diagnosis of asthma at the index visit or within the prior 6 months (yes or no), site of care (ED, urgent care, or outpatient clinic), occurrence of the index visit during influenza season (December 1 to March 31; yes or no), any laboratory test (yes or no) performed at the index visit, and any chest imaging (yes or no) at the index visit. We performed exact matching on age group, race and ethnicity, presence of a complex chronic condition, and site of care. We considered the social constructs of race and ethnicity because of their known association with antibiotic treatment of pneumonia and other conditions.^19,20,21,22^ Complex chronic conditions included those expected to last at least 12 months or result in hospitalization at a tertiary care center.^18^ We chose to study children with complex chronic conditions affecting any body system, as both children with respiratory conditions (eg, chronic lung disease) and conditions affecting other systems (eg, complex congenital heart disease) may be at increased risk of treatment with antibiotics and/or adverse outcomes.^23^ Laboratory tests included a complete blood cell count, blood culture, and measurement of C-reactive protein, blood gas, and lactic acid levels, as these tests may be more frequently performed in children with more severe disease.^24^ Chest imaging included chest radiography, computed tomography, or ultrasonography. In the propensity score–matched cohort, we calculated risk differences to compare outcomes across exposure groups and used the risk differences to calculate the number needed to prevent each outcome.

We performed 2 sensitivity analyses. First, we extended the exposure window of 0 to 1 days to a window of 0 to 2 days for antibiotic receipt. In this analysis, we measured outcomes from 3 to 14 days after the index visit. We did this because there is no validated definition of immediate antibiotic treatment, and it is possible that some antibiotics prescribed at the index visits—particularly for children with visits later in the day—could be reasonably dispensed 2 calendar days after the index visit. Second, we excluded children with a diagnosis of asthma or bronchiolitis, as children with bronchiolitis are more likely to have a viral etiology, and outcomes for children with these codiagnoses may be different from those for children without these conditions independent of the severity of the pneumonia or whether antibiotics were received.

Statistical significance was considered at 2-sided *P* < .05. Analyses were performed using SAS, version 9.4 (SAS Institute Inc).

## Results

Among the 103 854 eligible children with pneumonia included, the median age was 5 (IQR, 2-9) years; 49 189 (47.4%) were female and 54 665 (52.6%) were male. In addition, 8586 children (8.3%) were Hispanic; 27 622 (26.6%), non-Hispanic Black; 50 221 (48.4%), non-Hispanic White; 4388 (4.2%), other; and 13 055 (12.6%), missing. Outpatient clinics were the site of care in 66 098 index visits (63.6%). Asthma was present in 21 109 children (20.3%) and complex chronic conditions, in 8136 (7.8%) (Table 1).

**Table 1.** Characteristics of Children With Pneumonia, Stratified by Whether Antibiotics Were Dispensed

Characteristic	No. (%) of encounters		
	Overall (N = 103 854)	Antibiotics not dispensed (n = 20 435 [19.7%])	Antibiotics dispensed (n = 83 419 [80.3%])
Age, median (IQR), y	5 (2-9)	4 (2-8)	5 (3-9)
Age group, y			
1-4	47 633 (45.9)	11 158 (54.6)	36 475 (43.7)
5-12	43 877 (42.2)	7103 (34.8)	36 774 (44.1)
13-18	12 344 (11.9)	2174 (10.6)	10 170 (12.2)
Sex			
Female	49 189 (47.4)	9525 (46.6)	39 664 (47.5)
Male	54 665 (52.6)	10 910 (53.4)	43 755 (52.5)
Race and ethnicity			
Hispanic	8568 (8.3)	1493 (7.3)	7075 (8.5)
Non-Hispanic Black	27 622 (26.6)	5806 (28.4)	21 816 (26.2)
Non-Hispanic White	50 221 (48.4)	9199 (45.0)	41 022 (49.2)
Other^a^	4388 (4.2)	896 (4.4)	3492 (4.2)
Missing	13 055 (12.6)	3041 (14.9)	10 014 (12.0)
Complex chronic condition^b^	8136 (7.8)	2692 (13.2)	5444 (6.5)
Asthma^b^	21 109 (20.3)	4812 (23.5)	16 297 (19.5)
Site of care			
ED	32 934 (31.7)	8438 (41.3)	24 496 (29.4)
Urgent care	4822 (4.6)	545 (2.7)	4277 (5.1)
Outpatient clinic	66 098 (63.6)	11 452 (56.0)	54 646 (65.5)
Visit during influenza season (December 1 to March 31)	43 993 (42.4)	9192 (45.0)	34 801 (41.7)
Testing performed at the index visit			
Blood gas analysis	151 (0.1)	86 (0.4)	65 (0.1)
Complete blood cell count	7434 (7.2)	2193 (10.7)	5241 (6.3)
Blood culture	5412 (5.2)	1547 (7.6)	3865 (4.6)
C-reactive protein level	1188 (1.1)	419 (2.1)	769 (0.9)
Lactic acid level	430 (0.4)	179 (0.9)	251 (0.3)
Any of the above	10 419 (10.0)	2847 (13.9)	7572 (9.1)
Imaging performed at index visit	22 403 (21.6)	5225 (25.6)	17 178 (20.6)

Abbreviation: ED, emergency department.

^a^ Includes children of non-Hispanic ethnicities and race other than Black or White.

^b^ Diagnosed at index visit or within the past 6 months.

### Antibiotic Receipt

Among all children, 83 419 (80.3%) received antibiotics and 20 435 (19.7%) did not. Children aged 1 to 4 years had the lowest proportion of antibiotic receipt (36 475 of 47 633 [76.6%] received antibiotics) among all age groups. Non-Hispanic Black children received antibiotics at a lower frequency (21 816 of 27 622 [79.0%]) than non-Hispanic White children (41 022 of 50 221 [81.7%]) and Hispanic children (7075 of 8568 [82.6%]) (*P* < .001). Children visiting urgent care centers (4277 of 4822 [88.7%]) and outpatient clinics (54 646 of 66 098 [82.7%]) received antibiotics more frequently than those visiting EDs (24 496 of 32 934 [74.4%]) (*P* < .001). Children with complex chronic conditions received antibiotics after 5444 of 8136 encounters (66.9%).

### Outcomes

In unadjusted analysis, treatment failure occurred in 2204 children (10.8%) who did not receive antibiotics and 6878 (8.2%) who did (risk difference, 2.54 [95% CI, 2.08-3.00] percentage points) (eTable 2 in Supplement 1). Severe outcomes occurred in 234 children (1.1%) who did not receive antibiotics and in 450 (0.5%) who did (risk difference, 0.61 [95% CI, 0.45-0.76] percentage points).

After propensity score matching, 40 454 children were included in the adjusted analysis (20 277 children per exposure group). The propensity score–matched cohort was balanced on all variables included in the propensity score (eTable 3 in Supplement 1). In the propensity score–matched cohort, treatment failure occurred in 2167 children (10.7%) who did not receive antibiotics and in 1766 (8.7%) who did (risk difference, 1.98 [95% CI, 1.41-2.56] percentage points) (Table 2). The most common individual component of the treatment failure outcome was a new dispensed antibiotic prescription with a same-day ambulatory care visit, which occurred in 1783 children (8.8%) who did not receive antibiotics and in 1422 (7.0%) who did (risk difference, 1.78 [95% CI, 1.26-2.31] percentage points). Severe outcomes occurred in 230 children (1.1%) who did not receive antibiotics and in 133 (0.7%) who did (risk difference, 0.46 [95% CI, 0.28-0.64] percentage points).

**Table 2.** Risk of Treatment Failure and Other Clinical Outcomes in the Propensity Score-Matched Cohort

Outcomes^a^	No. (%) of encounters			Risk difference (95% CI), percentage points
	Overall (n = 40 454)	Antibiotics not dispensed (n = 20 277)	Antibiotics dispensed (n = 20 277)	
Treatment failure	3933 (9.7)	2167 (10.7)	1766 (8.7)	1.98 (1.41 to 2.56)
Hospitalization with a diagnosis of pneumonia	359 (0.9)	226 (1.1)	133 (0.7)	0.46 (0.28 to 0.64)
Visits to urgent care or the ED with a diagnosis of pneumonia	712 (1.8)	353 (1.7)	359 (1.8)	−0.03 (−0.28 to 0.22)
Antibiotic fill with same-day ambulatory visit^b^	3205 (7.9)	1783 (8.8)	1422 (7.0)	1.78 (1.26 to 2.31)
Complicated pneumonia	45 (0.1)	33 (0.2)	12 (0.1)	0.10 (0.04 to 0.17)
Severe outcome^c^	363 (0.9)	230 (1.1)	133 (0.7)	0.48 (0.30 to 0.66)
Other outcomes				
All-cause hospitalizations	519 (1.3)	344 (1.7)	175 (0.9)	0.84 (0.62 to 1.06)
All-cause visits to urgent care or the ED	2922 (7.2)	1510 (7.4)	1412 (7.0)	0.48 (−0.02 to 0.99)
Any antibiotic fill	6068 (15.0)	4174 (20.6)	1894 (9.3)	11.27 (10.58 to 11.96)

Abbreviation: ED, emergency department.

^a^ Outcomes were measured during the 2 to 14 days after the index visit.

^b^ Ambulatory visits included visits to offices, urgent care centers, or emergency departments.

^c^ Severe outcomes included hospitalization for pneumonia or a diagnosis of complicated pneumonia.

Severe outcomes occurred after a mean (SD) of 3.9 (2.7) days. Among children with severe outcomes, the median age was 4 (IQR, 1-7) years; 232 (33.9%) had a complex chronic condition; and 384 (56.2%) were initially evaluated in an ED. There were no significant differences in these characteristics between those who received antibiotics and those who did not.

### Sensitivity Analyses

In the sensitivity analysis that extended the exposure window for antibiotic receipt from 0 to 1 day to a window of 0 to 2 days, treatment failure occurred in 1358 children (7.3%) who did not receive antibiotics and in 1314 (7.1%) who did (risk difference, 0.24 [95% CI, −0.29 to 0.76] percentage points). Severe outcomes occurred after visits for 115 children (0.6%) who did not receive antibiotics and 80 (0.4%) who did (risk difference, 0.19 [95% CI, 0.04-0.33] percentage points) (eTable 4 in Supplement 1).

In the sensitivity analysis that excluded children with asthma and bronchiolitis, treatment failure occurred in 1536 children (9.9%) who did not receive antibiotics and 1397 (9.0%) who did (risk difference, 0.90 [95% CI, 0.25-1.54] percentage points). Severe outcomes occurred in 160 children (1.0%) who did not receive antibiotics and 106 (0.7%) who did (risk difference, 0.35 [95% CI, 0.14-0.55] percentage points).

## Discussion

In this multistate cohort study of insurance claims from publicly insured children with pneumonia discharged from ambulatory care settings, 1 in 5 children did not receive a prescription for antibiotics. Treatment failure was uncommon and severe outcomes were rare, occurring in approximately 10% and 1% of all children, respectively. Antibiotic treatment was associated with an approximately 2–percentage point risk difference for treatment failure and 0.5–percentage point risk difference for severe outcomes. In sensitivity analyses that considered a longer exposure window for antibiotic treatment or excluded children with asthma and bronchiolitis, risk differences were less than 1 percentage point.

Prior national studies^1,5,6^ have described rates of antibiotic prescribing for children with pneumonia that are similar to those identified in this study. A key innovation of our study that expands on prior findings is the identification of the frequency with which antibiotic prescriptions are received by patients, which—compared with antibiotic prescribing—is a measure that more closely reflects actual antibiotic use. The finding that a substantial proportion (19.7%) of children with pneumonia did not receive—and thus were not treated with—antibiotics allowed us to evaluate the effectiveness of antibiotics in children with pneumonia in clinical practice and emphasizes the importance of identifying groups of children who can safely be treated without antibiotics.

In addition to identifying the rate of antibiotic receipt, we found differences in the proportion of children who received antibiotics across demographic and clinical characteristics. While some of these differences (eg, in children with complex chronic conditions and asthma) may reflect different clinical presentations, others reflect opportunities to standardize care. For example, we observed that children discharged from the ED filled antibiotics at a substantially lower rate than those discharged from urgent care centers or office-based settings. This could be explained by different practices of assigning the diagnosis of pneumonia,^25^ different patterns of antibiotic prescribing,^26^ differences in parental expectations for antibiotics, or variable resources to fill antibiotic prescriptions among children who visit different settings. Further prospective investigation is needed to better understand these observed differences.

We also identified modest variability in the rates of filling antibiotic prescriptions according to the social constructs of race and ethnicity. Prior local and regional work has highlighted differences in rates of medication filling for children from minoritized racial or ethnic groups with a variety of medical conditions, including pneumonia.^20,21,22,27,28,29^ These findings reflect differences in both rates of antibiotic prescribing and rates of filling antibiotic prescriptions after they are prescribed. Like many of these studies, our study found that non-Hispanic Black children were less likely to fill a prescription than children of other races and ethnicities. Although the risk difference was modest in our study, the results—together with prior similar findings—imply an urgent need to reduce inequities in antibiotic treatment.

Our findings that 8.7% of children who received antibiotics experienced treatment failure and 0.7% experienced severe outcomes are similar to findings from prior cohort studies and clinical trials of children receiving antibiotic treatment for pneumonia,^30,31,32^ allowing for differences in the definition of treatment failure across studies. A strength of our study is that, unlike these prior studies, we evaluated outcomes in children who did not receive antibiotics. In this way, this study investigates a key clinical question that has not yet been thoroughly investigated—whether antibiotics are necessary in all children with pneumonia managed in an outpatient setting, in whom viruses are a common cause. We were reassured that new antibiotics dispensed were associated with most treatment failures and that the risk of severe outcomes was small, regardless of whether children did (0.7%) or did not (1.1%) receive antibiotics, even after propensity score matching to account for baseline characteristics.

We identified modest differences between those treated and not treated with antibiotics, whereby those not treated with antibiotics had a 1.78% statistically greater absolute risk of treatment failure. Put differently, the number needed to treat with antibiotics was 50 children to prevent a treatment failure by our definition. This finding was largely explained by differences in the dispensation of new antibiotic prescriptions rather than by severe outcomes, for which the number needed to treat was 250 children. Additionally, the risks of treatment failure in those who did not vs did receive antibiotics were reduced after excluding children with asthma or bronchiolitis (9.9% vs 9.0%) and after lengthening the exposure window (7.3% vs 7.1%), which suggests that some of the differences in outcomes may be explained by comorbid lower respiratory tract illnesses, the specific definition of the exposure window, or misclassification of the diagnosis of pneumonia. Together, these results suggest that most children do not experience treatment failure or severe outcomes if they do not receive antibiotics. Prospective studies are warranted to determine which children with pneumonia can be safely treated without antibiotics.

### Limitations

We acknowledge the limitations of this study. First, we included only Medicaid-insured children. As a result, our findings may not generalize to children with other forms of insurance. Although we do not expect insurance status to affect underlying biological phenomena, differences in access to care according to insurance status could result in distinct outcomes. Second, the database did not allow us to distinguish visits in which antibiotics were prescribed but not dispensed from those in which antibiotics were not prescribed. Although full information about antibiotic dispensation allowed for improved definition of the exposure in the comparative effectiveness analysis, we were unable to perform an intention-to-treat analysis according to whether antibiotics were prescribed. Third, we were unable to confirm that the dispensed antibiotics were prescribed at the index visit rather than prior to the index visit for a different indication or by telephone after the index visit. We attempted to mitigate misclassification bias by including only antibiotics that are recommended for treatment of pneumonia, by defining an exposure window according to the time distribution of antibiotic filling, and by performing a sensitivity analysis with a longer exposure window. Fourth, we could not confirm that antibiotics were consumed. To the extent that children received but did not take medications as prescribed, there may be misclassification bias. Fifth, certain clinical and demographic data were not available in the claims data. Although we attempted to mitigate potential confounding by indication using a propensity score–matched cohort that was balanced on baseline characteristics, it is possible that we did not fully account for unmeasured differences in clinical presentations (eg, vital signs) or other characteristics (eg, vaccination status, unmeasured comorbidities). Sixth, we did not have access to microbiological data to understand which pneumonias were bacterial or viral. However, the study reflects clinical practice, where an etiology is rarely determined. Seventh, we acknowledge that some children with pneumonia may not have been assigned a diagnosis code for pneumonia. Because we included patients based on administrative codes for pneumonia, our findings should not be applied to other children. Eighth, because we calculated risk differences for treatment failure in the entire propensity score–matched cohort, we cannot comment on how the risk difference might vary according to age group or other patient characteristics. Future studies should seek to identify subgroups of children with pneumonia for whom the absolute risk and/or the risk difference for treatment failure is particularly low. Finally, we were unable to measure differences in symptoms across exposure groups during the follow-up period. Future prospective studies should consider these important patient-centered clinical outcomes.

## Conclusions

In this cohort study, approximately 20% of publicly insured children discharged from ambulatory care settings with a diagnosis of pneumonia did not receive antibiotics. Given that this constitutes a substantial proportion of children with pneumonia, the observed differences in treatment failure were modest, and severe outcomes were rare regardless of antibiotic treatment, future research should determine which children with pneumonia can be safely and effectively treated without immediate antibiotic treatment.

## Notes

Supplement 1. **eTable 1.** Administrative Codes **eTable 2.** Unadjusted Risk of Treatment Failure and Other Clinical Outcomes (N = 103 854), Stratified by Whether Antibiotics Were Dispensed **eTable 3.** Characteristics of the Propensity Score–Matched Cohort **eTable 4.** Sensitivity Analysis of Outcomes After Changing the Exposure Window for Antibiotic Treatment From 1 to 2 Days **eTable 5.** Sensitivity Analysis of Outcomes After Exclusion of Children With Bronchiolitis or Asthma

Supplement 2.
Data Sharing Statement
