# Supplementary material for: Outpatient Antibiotic Use and Treatment Failure Among Children With Pneumonia
Source: JAMA Netw Open. 2024 Oct 29;7(10):e2441821. doi: 10.1001/jamanetworkopen.2024.41821 (PMC11522934; doi:10.1001/jamanetworkopen.2024.41821)
Supplement: Supplement 1. — eTable 1. Administrative Codes eTable 2. Unadjusted Risk of Treatment Failure and Other Clinical Outcomes (N = 103 854), Stratified by Whether Antibiotics Were Dispensed eTable 3. Characteristics of the Propensity Score–Matched Cohort eTable 4. Sensitivity Analysis of Outcomes After Changing the Exposure Window for Antibiotic Treatment From 1 to 2 Days eTable 5. Sensitivity Analysis of Outcomes After Exclusion of Children With Bronchiolitis or Asthma [file jamanetwopen-e2441821-s001.pdf]

Supplementary Online Content

Shapiro DJ, Hall M, Neuman MI, et al. Outpatient antibiotic use and treatment failure among children with pneumonia. *JAMA Netw Open*. 2024;7(10):e2441821. doi:10.1001/jamanetworkopen.2024.41821

eTable 1. Administrative Codes

eTable 2. Unadjusted Risk of Treatment Failure and Other Clinical Outcomes (N = 103 854), Stratified by Whether Antibiotics Were Dispensed

eTable 3. Characteristics of the Propensity Score–Matched Cohort

eTable 4. Sensitivity Analysis of Outcomes After Changing the Exposure Window for Antibiotic Treatment From 1 to 2 Days

eTable 5. Sensitivity Analysis of Outcomes After Exclusion of Children With Bronchiolitis or Asthma

This supplementary material has been provided by the authors to give readers additional information about their work.

© 2024 Shapiro DJ et al. *JAMA Network Open*.

eTable 1. Administrative Codes

Description	Code type[1]	Values
Pneumonia diagnosis	ICD-10	J13, J14, J15, J18
Asthma diagnosis	ICD-10	493.00, 493.01, 493.02, 493.10, 493.11, 493.12, 493.20, 493.21, 493.22, 493.81, 493.82, 493.90, 493.91, 493.92, J45.20, J45.21, J45.22, J45.30, J45.31, J45.32, J45.40, J45.41, J45.42, J45.50, J45.51, J45.52, J459.01, J459.02, J459.09, J459.90, J459.91, J459.98
Bronchiolitis diagnosis	ICD-10	J21.0, J21.1, J21.8, J21.9
Concomitant bacterial infections	ICD-10	J01.00, J01.01, J01.10, J01.11, J01.20, 0J01.40, J01.41, J01.80, J01.81, J01.90 J01.91, J02.0, J03.00, J03.01, J04.10, J05.10, J11.83, J2.00, J2.01, J2.02, J3.20, J3.21, J3.22, J32.4, J32.8, J32.9, J36, J85.0, J85.1, J85.2, J86.0, J86.9, J95.851, J98.51, K04.7, K35.3, K35.80, K35.89, K37, K81.0, K83.09, L01.00, L01.01, L01.02, L01.03, L01.09, L02.11, L02.31, L02.32, L02.416, L02.434, L02.512, L02.811, L02.91, L03.011, L03.012, L03.019, L03.031, L03.032, L03.039, L03.113, L03.115, L03.116, L03.119, L03.213, L03.312, L03.313, L03.317, L03.811, L03.90, L03.91, L04.0, L04.9, L08.0, L08.89, L08.9, M60.009, M60.89, M60.9, N10, N30.00, N30.01, N30.80, N30.90, N30.91, N34.2, N39.0, N61.0, R65.21, R78.81, S518.52A
Antibiotic fills	NDC	Codes for the following with oral route of administration: amoxicillin, amoxicillin/clavulanate, azithromycin, cefaclor, cefadroxil, cefdinir, cefixime, cefpodoxime, cefprozil, cefuroxime, cephalexin, ciprofloxacin, ciprofloxacin, clarithromycin, clindamycin, doxycycline, erythromycin, levofloxacin, linezolid, penicillin
Complicated pneumonia	ICD-10, ICD-10 Procedure	ICD-10: J85.0, J85.1, J85.2, J86.0, J86.9, J90, J91.8, J94.1, J94.8, J94.9, R09.1, ICD-10 procedure: 0B9N00Z, 0B9N0ZZ, 0B9N30Z, 0B9N3ZZ, 0B9N40Z, 0B9N4ZZ, 0B9N80Z, 0B9N8ZZ, 0B9P00Z, 0B9P0ZZ, 0B9P30Z, 0B9P3ZZ, 0B9P40Z, 0B9P4ZZ, 0B9P80Z, 0B9P8ZZ, 0BCN0ZZ, 0BCN3ZZ, 0BCN4ZZ, 0BCP0ZZ, 0BCP3ZZ, 0BCP4ZZ, 0BHQ0YZ, 0BHQ3YZ, 0BHQ4YZ, 0BHQ7YZ, 0BHQ8YZ, 0BJ04ZZ, 0BJK4ZZ, 0BJL4ZZ, 0BJQ0ZZ, 0BJQ3ZZ, 0BJQ4ZZ, 0BJQ7ZZ, 0BJQ8ZZ, 0BJQXZZ, 0W9900Z, 0W990ZZ, 0W9930Z, 0W993ZZ, 0W9940Z, 0W994ZZ, 0W9B00Z, 0W9B0ZZ, 0W9B30Z, 0W9B3ZZ, 0W9B40Z, 0W9B4ZZ, 0WC90ZZ, 0WC93ZZ, 0WC94ZZ, 0WCB0ZZ, 0WCB3ZZ, 0WCB4ZZ, 0WCQ0ZZ, 0WJ90ZZ, 0WJ94ZZ, 0WJB0ZZ, 0WJB4ZZ, 0WJC0ZZ, 0WJC3ZZ, 0WJC4ZZ, 0WJQ0ZZ, 0WJQ4ZZ, 0WJQ7ZZ

[1] ICD-10: International Classification of diseases, 10th revision; NDC: National Drug Code

© 2024 Shapiro DJ et al. *JAMA Network Open*.

eTable 2. Unadjusted Risk of Treatment Failure and Other Clinical Outcomes (N = 103 854), Stratified by Whether Antibiotics Were Dispensed

Outcomes[1]	Overall, N (%)	Antibiotics not filled, N (%)	Antibiotics filled, N (%)	Risk Difference (95% CI)
**Treatment failure**	9,082 (8.7)	2,204 (10.8)	6,878 (8.2)	2.54 (2.08, 3.00)
Hospitalization with a diagnosis of pneumonia	678 (0.7)	230 (1.1)	448 (0.5)	0.59 (0.44, 0.74)
Visits to urgent care or the emergency department with a diagnosis of pneumonia	1,974 (1.9)	356 (1.7)	1,618 (1.9)	-0.20 (-0.40, 0.00)
Antibiotic fill with same-day ambulatory visit[2]	7,325 (7.1)	1,817 (8.9)	5,508 (6.6)	2.29 (1.86, 2.71)
Complicated pneumonia	95 (0.1)	34 (0.2)	61 (0.1)	0.09 (0.03, 0.15)
**Severe outcome[3]**	684 (0.7)	234 (1.1)	450 (0.5)	0.61 (0.45, 0.76)
**Other outcomes**				
All-cause hospitalizations	966 (0.9)	354 (1.7)	612 (0.7)	1.00 (0.81, 1.19)
All-cause visits to urgent care or the emergency department	7,025 (6.8)	1,520 (7.4)	5,505 (6.6)	0.84 (0.44, 1.24)
Any antibiotic fill	11,670 (11.2)	4,230 (20.7)	7,440 (8.9)	11.78 (11.19, 12.37)

[1] Outcomes were measured during the 2-14 days after the index visit.

[2] Ambulatory visits included visits to offices, urgent care centers, or emergency rooms.

[3] Severe outcomes included hospitalization for pneumonia or a diagnosis of complicated pneumonia.

© 2024 Shapiro DJ et al. *JAMA Network Open*.

eTable 3. Characteristics of the Propensity Score–Matched Cohort

Characteristic	Overall, N (%)	Antibiotics not filled, N (%)	Antibiotics filled, N (%)	Standardized Difference	p-value
Number of encounters	40,454	20,227 (50.0)	20,227 (50.0)		
Age, in years, median (IQR)	4 [2, 8]	4 [2, 8]	4 [2, 8]	0.01	1.00
Age group (years)				0.00	1.00
1-4	22,116 (54.7)	11,058 (54.7)	11,058 (54.7)		
5-12	14,092 (34.8)	7,046 (34.8)	7,046 (34.8)		
13-18	4,246 (10.5)	2,123 (10.5)	2,123 (10.5)		
Female sex	18847 (46.6)	9430 (46.6)	9417 (46.6)	0.00	0.90
Race and ethnicity				0.00	1.00
Non-Hispanic White	18,306 (45.3)	9,153 (45.3)	9,153 (45.3)		
Non-Hispanic Black	11,520 (28.5)	5,760 (28.5)	5,760 (28.5)		
Hispanic	2,944 (7.3)	1,472 (7.3)	1,472 (7.3)		
Other	1,712 (4.2)	856 (4.2)	856 (4.2)		
Missing	5,972 (14.8)	2,986 (14.8)	2,986 (14.8)		
Complex chronic condition[1]	5,068 (12.5)	2,534 (12.5)	2,534 (12.5)	0.00	1.00
Asthma[1]	9,398 (23.2)	4,699 (23.2)	4,699 (23.2)	0.00	1.00
Site of care				0.00	1.00
Emergency Department	16,720 (41.3)	8,360 (41.3)	8,360 (41.3)		
Urgent Care	1,042 (2.6)	521 (2.6)	521 (2.6)		
Outpatient Clinic	22,692 (56.1)	11,346 (56.1)	11,346 (56.1)		
Influenza season (December – March)	18,196 (45.0)	9,098 (45.0)	9,098 (45.0)	0.00	1.00
Lab testing performed at the index visit	5,362 (13.3)	2,681 (13.3)	2,681 (13.3)	0.00	1.00
Imaging performed at index visit	10,254 (25.3)	5,127 (25.3)	5127 (25.3)	0.00	1.00

[1]Diagnosed in the prior 6 months

© 2024 Shapiro DJ et al. *JAMA Network Open*.

eTable 4. Sensitivity Analysis of Outcomes After Changing the Exposure Window for Antibiotic Treatment From 1 to 2 Days

Outcomes[1]	Overall, N (%)	Antibiotics not dispensed, N (%)	Antibiotics dispensed, N (%)	Risk Difference (95% CI)
**Treatment failure**	2,672 (7.2)	1,358 (7.3)	1,314 (7.1)	0.24 (-0.29, 0.76)
Hospitalization with a diagnosis of pneumonia	192 (0.5)	112 (0.6)	80 (0.4)	0.17 (0.03, 0.32)
Visits to urgent care or the emergency department with a diagnosis of pneumonia	477 (1.3)	224 (1.2)	253 (1.4)	-0.16 (-0.38, 0.07)
Antibiotic fill with same-day ambulatory visit[2]	2,211 (5.9)	1,123 (6.0)	1,088 (5.8)	0.19 (-0.29, 0.67)
Complicated pneumonia	30 (0.1)	19 (0.1)	11 (0.1)	0.04 (-0.01, 0.10)
**Severe outcome[3]**	195 (0.5)	115 (0.6)	80 (0.4)	0.19 (0.04, 0.33)
**Other outcomes**				
All-cause hospitalizations	308 (0.8)	194 (1.0)	114 (0.6)	0.43 (0.25, 0.61)
All-cause visits to urgent care or the emergency department	2,215 (6.0)	1,133 (6.1)	1,082 (5.8)	0.27 (-0.21, 0.75)
Any antibiotic fill	3,934 (10.6)	2,444 (13.1)	1,490 (8.0)	5.13 (4.50, 5.75)

[1] Outcomes were measured during the 3-14 days after the index visit.

[2] Ambulatory visits included visits to offices, urgent care centers, or emergency rooms.

[3] Severe outcomes included hospitalization for pneumonia or a diagnosis of complicated pneumonia.

© 2024 Shapiro DJ et al. *JAMA Network Open*.

eTable 5. Sensitivity Analysis of Outcomes After Exclusion of Children With Bronchiolitis or Asthma

**Outcomes**[1]	**Overall, N (%)**	**Antibiotics not dispensed, N (%)**	**Antibiotics dispensed, N (%)**	**Risk Difference (95% CI)**
**Treatment failure**	2,933 (9.4)	1,536 (9.9)	1,397 (9.0)	0.90 (0.25, 1.54)
Hospitalization with a diagnosis of pneumonia	262 (0.8)	156 (1.0)	106 (0.7)	0.32 (0.12, 0.53)
Visits to urgent care or the emergency department with a diagnosis of pneumonia	590 (1.9)	280 (1.8)	310 (2.0)	-0.19 (-0.50, 0.11)
Antibiotic fill with same-day ambulatory visit[2]	2,354 (7.6)	1,245 (8.0)	1,109 (7.1)	0.88 (0.29, 1.47)
Complicated pneumonia	39 (0.1)	27 (0.2)	12 (0.1)	0.1 (0.02, 0.18)
**Severe outcome**[3]	266 (0.9)	160 (1)	106 (0.7)	0.35 (0.14, 0.55)
**Other outcomes**				
All-cause hospitalizations	388 (1.2)	246 (1.6)	142 (0.9)	0.67 (0.42, 0.91)
All-cause visits to urgent care or the emergency department	2,260 (7.3)	1,150 (7.4)	1,110 (7.1)	0.26 (-0.32, 0.83)
Any antibiotic fill	4,443 (14.3)	2,975 (19.2)	1,468 (9.5)	9.71 (8.93, 10.48)

[1] Outcomes were measured during the 2-14 days after the index visit.

[2] Ambulatory visits included visits to offices, urgent care centers, or emergency rooms.

[3] Severe outcomes included hospitalization for pneumonia or a diagnosis of complicated pneumonia.

© 2024 Shapiro DJ et al. *JAMA Network Open*.
