# Supplementary material for: Outpatient Antibiotic Use and Treatment Failure Among Children With Pneumonia
Source: JAMA Netw Open. 2024 Oct 29;7(10):e2441821. doi: 10.1001/jamanetworkopen.2024.41821 (PMC11522934; doi:10.1001/jamanetworkopen.2024.41821)
Supplement: Supplement 2. — Data Sharing Statement [file jamanetwopen-e2441821-s002.pdf]

Data Sharing Statement

Shapiro. Outpatient Antibiotic Use and Treatment Failure Among Children With Pneumonia. *JAMA Netw Open*. Published October 29, 2024. doi:10.1001/jamanetworkopen.2024.41821

Data
Data available: No

Additional Information
Explanation for why data not available: MarketScan data are proprietary.
